# Editorial: New insight into immune cells in the development of non-traumatic osteonecrosis of the femoral head

**DOI:** 10.3389/fcell.2024.1388531

**Published:** 2024-05-07

**Authors:** Yu Zhou, Jinhui Ma, Qingyu Zhang, Bailiang Wang

**Affiliations:** ^1^ Department of Orthopaedic Surgery, Peking University China-Japan Friendship School of Clinical Medicine, Beijing, China; ^2^ Department of Orthopaedic Surgery, Center for Osteonecrosis and Joint Preserving and Reconstruction, China-Japan Friendship Hospital, Beijing, China; ^3^ Department of Orthopaedic Surgery, Shandong Provincial Hospital Affiliated to Shandong First Medical University, Jinan, China

**Keywords:** osteonecrosis of femoral head, osteoimmunity, immune cells, pathogenesis, macrophages

Osteonecrosis of the femoral head (ONFH) is a challenging condition characterized by compromised subchondral microcirculation and osteocyte apoptosis (Mont et al., 2020). Currently, the pathogenesis of ONFH remains incompletely elucidated, but recent researches highlight the crucial role of immune factors in the onset and progression of this condition. Under normal circumstances, a balance is maintained between osteoblasts and osteoclasts, with immune cells playing a significant role in preserving this equilibrium. When ONFH occurs, locally damaged cells release a substantial amount of chemotactic and cytokine factors, disrupting immune balance. The growth factor-cytokine imbalance needs to be corrected. If not corrected, osteoclasts will become more active than osteoblasts over time, which would make bone repair difficult and eventually lead to irreversible collapse of the femoral head. This research project comprises four articles that delve deeper and strengthen our understanding of non-traumatic femoral head necrosis (Ma et al., 2019; Wang et al., 2021).

With the evolution of microfluidic systems, organ-on-chip has become a trending Research Topic. Fan et al. established a robust microfluidic platform of bone on-a-chip, successfully achieving multi-component cultures of bone microvascular endothelial cells (BMEC), human lung fibroblasts, and hydroxyapatite. Through this technology, they investigated changes in TNF-alpha levels during the occurrence of ONFH and its impact on bone microvascular endothelial cells. Notably, they also innovatively constructed and improved DNA adapters for TNF-alpha, confirming its effectiveness in the treatment of ONFH and paving the way for a new Frontier in early intervention for this condition.

Femoral head collapse is a crucial signal of the deterioration of ONFH, associated with alterations in the trabecular structure within the femoral head. He et al. employed techniques such as immunohistochemistry, micro-CT, nanoindentation, acid-etched scanning electron microscopy, and others to assess and compare the ultrastructural changes in bone cell morphology and nanomechanical features in different regions of necrotic femoral heads. Their research findings are of paramount importance for our understanding of the microstructural and macro-mechanical alterations in the progression of femoral head necrosis.

Currently, magnetic resonance imaging remains the gold standard for early diagnosis of femoral head necrosis, but it is expensive and time-consuming. Yang et al. utilized Phase-contrast imaging (PCI) with synchrotron hard X-ray to observe the impact of icariin, the primary bioactive compound found in the traditional Chinese herb Epimedium brevicornum known for “strengthening bone and tonifying kidney,” on the early glucocorticoid-induced osteonecrosis of the femoral head rabbit model. The reliability of this technique was confirmed through bone tissue morphological observations and HE staining. PCI with synchrotron hard X-ray demonstrated the capability to visualize the microstructure of the femoral head, which is comparable to Micro-CT and HE staining. It could potentially serve as a non-invasive alternative for histological examination in the early diagnosis of ONFH.

Whole body vibration therapy (WBVT) is a novel form of physical treatment that can stimulate bone formation, and its therapeutic effect on osteoporosis has been validated. The research of Tian et al. examined the therapeutic potential of WBVT in a rat model of ONFH, revealing that it significantly improves the structure of subchondral trabeculae in the femoral heads by activating Piezo1. Notably, they also proposed, for the first time, that WBVT may enhance blood supply of the femoral head through the activation of the Piezo1 pathway and the HIF-1a/VEGF axis, thereby impeding the progression of ONFH.

In summary, this Research Topic on *New Insight into Immune Cells in the Development of Non-Traumatic Osteonecrosis of the Femoral Head* encompasses four high-quality studies. Each article addresses different aspects of non-traumatic femoral head necrosis, providing crucial insights into the latest research advancements in pathophysiology, diagnosis, and treatment of ONFH. As our understanding of the pathophysiological mechanisms of femoral head necrosis deepens, we believe that overcoming this global challenge is undoubtedly achievable in the near future.
